# Dietary antioxidant seleno-L-methionine protects macrophages infected with *Burkholderia thailandensis*

**DOI:** 10.1371/journal.pone.0238174

**Published:** 2020-09-03

**Authors:** Michelle M. Pomposello, Kaitlyn Nemes, Kara Mosovsky

**Affiliations:** Department of Biological Sciences, Moravian College, Bethlehem, Pennsylvania, United States of America; J Craig Venter Institute, UNITED STATES

## Abstract

*Burkholderia pseudomallei* is a facultative intracellular pathogen and the causative agent of melioidosis, a potentially life-threatening disease endemic in Southeast Asia and Northern Australia. Treatment of melioidosis is a long and costly process and the pathogen is inherently resistant to several classes of antibiotics, therefore there is a need for new treatments that can help combat the pathogen. Previous work has shown that the combination of interferon-gamma, an immune system activator, and the antibiotic ceftazidime synergistically reduced the bacterial burden of RAW 264.7 macrophages that had been infected with either *B*. *pseudomallei* or *Burkholderia thailandensis*. The mechanism of the interaction was found to be partially dependent on interferon-gamma-induced production of reactive oxygen species inside the macrophages. To further confirm the role of reactive oxygen species in the effectiveness of the combination treatment, we investigated the impact of the antioxidant and reactive oxygen species scavenger, seleno-L-methionine, on intracellular and extracellular bacterial burden of the infected macrophages. In a dose-dependent manner, high concentrations of seleno-L-methionine (1000 μM) were protective towards infected macrophages, resulting in a reduction of bacteria, on its own, that exceeded the reduction caused by the antibiotic alone and rivaled the effect of ceftazidime and interferon-gamma combined. Seleno-L-methionine treatment also resulted in improved viability of infected macrophages compared to untreated controls. We show that the protective effect of seleno-L-methionine was partly due to its inhibition of bacterial growth. In summary, our study shows a role for high dose seleno-L-methionine to protect and treat macrophages infected with *B*. *thailandensis*.

## Introduction

*Burkholderia pseudomallei* is a Gram negative, facultative intracellular pathogen and the etiological agent of melioidosis [1], a life-threatening opportunistic infection mainly observed in South Asia, Northern Australia, and other tropical regions [1–3]. The bacteria can be isolated from soil and water sources and transmission to humans typically occurs through inhalation, ingestion, or through abrasions on the skin [1, 2]. *B*. *pseudomallei* can invade several cell types including phagocytes such as macrophages and neutrophils [4–7] and the details of its intracellular pathogenesis are well known. After uptake into the cell, it can escape into the cytoplasm [4] where it can replicate and polymerize host-cell actin to facilitate its spread from cell-to-cell thus creating multinucleated giant cells [1, 6, 8–10].

Treatment of melioidosis is a long and expensive process requiring both an early intravenous antibiotic phase and a lengthy eradication phase that together can last three months or more [1, 11]. Although there is a standard treatment for melioidosis, its prognosis is never guaranteed since *B*. *pseudomallei* is inherently resistant to antibiotics and possesses several virulence factors that aid in host evasion [3, 12–14]. Even with antibiotic therapy, mortality is high and patients can experience recurrent or chronic infections [3, 15]. Given the length of time required to treat melioidosis, the organism’s inherent antibiotic resistance and host-evasion strategies, as well as the potential for an expanding and changing distribution of melioidosis around the world, new therapies are needed [2, 3, 11–14, 16, 17].

Better understanding of the natural host response to melioidosis has helped inform research on potential therapies. In patients with melioidosis, increased levels of interferon-gamma (IFN-γ), a proinflammatory cytokine and macrophage activator, have been observed during the natural immune response against *B*. *pseudomallei* [18–20]. Studies show that IFN-γ is essential for host survival and clearance of bacterial burden [21–23] during melioidosis. Previous research has also shown a synergistic effect between this immune stimulant and antibiotics when treating macrophages infected with *B*. *pseudomallei* or *Burkholderia thailandensis*. In particular, IFN-γ and ceftazidime, an antibiotic used to treat melioidosis, both had individual effects in reducing bacterial burden from an *in vitro* macrophage infection model, but their combined effect resulted in synergistic reduction of bacterial burden [24, 25]. This effect was shown both *in vivo* [25] as well as *in vitro* through the use of several macrophage cell lines [24], suggesting that therapies that combine immune stimulation with antibiotics may be promising strategies for treating melioidosis. Evidence suggested that IFN-γ-stimulated production of reactive oxygen species (ROS) contributed to the success of the combination therapy, as the effect of IFN-γ was reversed by a dose-dependent addition of the ROS-scavenging cellular antioxidants glutathione (GSH) and N-acetylcysteine (NAC), a precursor to GSH [24]. Furthermore, L-buthionine sulfoximine, a pro-oxidant drug that inhibits GSH synthesis [26, 27], increased levels of intracellular ROS by itself, and similar to IFN-γ, combined with ceftazidime treatment to synergistically decrease the intracellular bacterial burden [24].

For this study, our aim was to corroborate the role of ROS in reducing *B*. *thailandensis* burden in infected macrophages using a third ROS-scavenging antioxidant, seleno-L-methionine (SeMet), an organic dietary antioxidant that feeds into similar metabolic pathways as NAC and GSH to produce glutathione peroxidases which help regulate the redox state of the cell [28]. For these studies we used *B*. *thailandensis*, a biosafety level 1 organism and a close relative and model organism for the study of *B*. *pseudomallei* [8, 9, 29]. With the addition of SeMet, we expected to see a reversal of the effects caused by IFN-γ and ceftazidime, similar to what we had observed for NAC and GSH. Instead we found that high concentrations of SeMet itself resulted in significantly reduced bacterial burden inside and outside of infected macrophages without impacting macrophage toxicity. The ability of SeMet to inhibit bacterial growth likely contributes to its protective effect on macrophages.

## Materials and methods

### Biochemicals

Ceftazidime hydrate and seleno-L-methionine (Sigma-Aldrich, St. Louis, MO) were dissolved in water, sterile filtered, and frozen in aliquots of 10 mg/ml and 50 mM solutions, respectively. IFN-γ (PeproTech, Rocky Hill, NJ) was reconstituted according to the manufacturer’s instructions, aliquoted (100 μg/ml) and frozen at -80°C until use. Other reagents included kanamycin monosulfate (MP Biomedicals, Solon, OH) and 0.4% Trypan Blue solution (Mediatech, Manassas, VA).

### Bacteria

*B*. *thailandensis* E264 was purchased from American Type Culture Collection (Manassas, VA) and used for all experiments. An isolated colony was inoculated into sterile tryptic soy broth and incubated at 37°C on a rotary shaker at 200 rpm for 16–18 hours. Bacterial stocks were then aliquoted and frozen at -80°C with 15–20% glycerol until needed. Single-use vials of bacteria were thawed and resuspended immediately before experimental use.

### Cell line

RAW 264.7 murine macrophages were purchased from American Type Culture Collection (Manassas, VA, catalogue #ATCC® TIB-71™, deposited by WC Raschke [30]) and used for all *in vitro* infection assays. Macrophages were grown in complete minimal essential media (cMEM) which was created using minimal essential media (Gibco, ThermoFisher Scientific, Waltham, MA) supplemented to a final concentration of 10% fetal bovine serum (Atlas, Fort Collins, CO), 2 mM L-glutamine (Gibco, ThermoFisher Scientific), 0.5X essential amino acids (Gibco, ThermoFisher Scientific), 0.1 mM non-essential amino acids (Gibco, ThermoFisher Scientific), and 9 mM sodium bicarbonate solution. The cell line was maintained in cMEM supplemented with 100 units/ml penicillin and 100 μg/ml streptomycin, while all experiments were performed in antibiotic-free cMEM. Cells were passaged when they reached ~80–90% confluency and were maintained in an incubator at 37°C with 5% CO_2_ and 80% relative humidity.

### Macrophage infection assay

Infection assays were performed as previously described [24, 25]. Macrophages were washed in phosphate buffered saline (PBS) and seeded into 24-well plates at a final concentration of 200,000 cells per well in 500 μl of antibiotic-free cMEM. The next day, the adherent macrophages were washed with 1 ml of PBS after which *B*. *thailandensis* was added to the macrophages at a multiplicity of infection of 5 and incubated for 1 hour at 37°C with 5% CO_2_. The infection solution was removed and the macrophages were washed once with 2 ml of PBS. The macrophages were then incubated with 1 ml of a 350 μg/ml kanamycin monosulfate solution for 1 hour to kill remaining extracellular bacteria. After two 2 ml washes with PBS, macrophages were treated with ceftazidime (10 μg/ml), IFN-γ (10 ng/ml), and/or SeMet (various concentrations) for 18 hours at a total volume of 500 μl per well in cMEM. After treatment, extracellular bacterial burden was quantified by gently resuspending the bacteria in the wells and plating 10-fold serial dilutions of the supernatants onto Luria-Bertani (LB) agar followed by colony counts 36–48 hours after incubation. To quantify intracellular bacterial burden, macrophages were first washed three times with 2 ml of PBS to remove extracellular bacteria and then lysed with 1ml of sterile distilled water for 5 minutes. After 5 minutes, the lysates were resuspended and transferred to a dilution plate containing PBS. Serial 10-fold dilutions of the lysates were plated on LB agar and colonies were counted 36–48 hours after incubation.

### Live/dead cell counts

Toxicity of SeMet on infected macrophages was assessed through trypan blue exclusion. Macrophages were seeded into 24-well plates and infected as described for the macrophage infection assay. Infected macrophages were treated with SeMet alone or in combination with other treatments at 37°C for 18 hours. After incubation, the cells were washed gently three times with 2 ml of PBS. Pictures of each of three triplicate wells were taken within 5 minutes of the addition of a 1:4 dilution of trypan blue solution to each well. Five equally spaced fields of view were imaged from each treated well, and live vs. dead cells were manually counted according to trypan blue exclusion principles. Images were captured using an Olympus® CKX53F inverted microscope fitted with an SC30 color camera through the Cellsens Entry® imaging software, version 1.14.

### Bacterial killing assay

In order to assess the effects of SeMet on bacteria alone, an in-vitro assay was performed with bacteria and varying concentrations of SeMet. The assay was performed in the absence of macrophages, but with otherwise similar culture conditions as the macrophage infection model. Bacteria were thawed and applied to wells with or without SeMet at a concentration of 1x10^6^ bacteria per well in cMEM and incubated at 37°C with 5% CO_2_ for 18 hours. Remaining bacteria were resuspended and serial dilutions were plated on LB agar followed by a 36–48 hour incubation. Bacterial densities, colony forming units (CFU)/ml, were calculated based on the number of colonies that grew. Slides of the remaining bacteria were also prepared, stained with crystal violet, and imaged with a Leica DM500 light microscope with 5-megapixel ICC50W camera and Leica AirLab App.

### Statistical analyses

Means, standard error of the mean (SEM), and statistical analyses were calculated and plotted using Prism® software version 7.0d (GraphPad, La Jolla, CA). For the statistical analysis of three or more averages, a one-way analysis of variance (ANOVA) was performed, followed by Tukey’s post-test for multiple comparisons. Statistically significant differences were noted for *P* values < 0.05.

## Results

### SeMet treatment of infected macrophages leads to significant reduction of intracellular and extracellular bacterial burden

We previously found that two cellular antioxidants and ROS pathway inhibitors, N-acetylcysteine (NAC) and glutathione (GSH), abolished the synergistic effect of IFN-γ and ceftazidime to reduce intracellular bacterial burden in *B*. *thailandensis* infected macrophages [24]. These results supported the role of IFN-γ-induced ROS in contributing to the reduction of bacteria in the combination treatment of macrophages. While GSH and NAC, a precursor to GSH, are both ROS-scavenging antioxidants themselves, they also contribute to the production of glutathione peroxidases which convert reactive hydrogen peroxides to non-toxic compounds (water and oxygen). SeMet is an important ROS-scavenging antioxidant on its own but also feeds into the same glutathione peroxidase pathways as GSH and NAC through incorporation of selenium into several important selenium-containing glutathione peroxidases [28, 31, 32]. Therefore we chose SeMet, an organic dietary antioxidant [33], as a third antioxidant to corroborate the role of ROS in the synergistic reduction of bacterial burden in macrophages treated with ceftazidime and IFN-γ.

**Fig 1:**
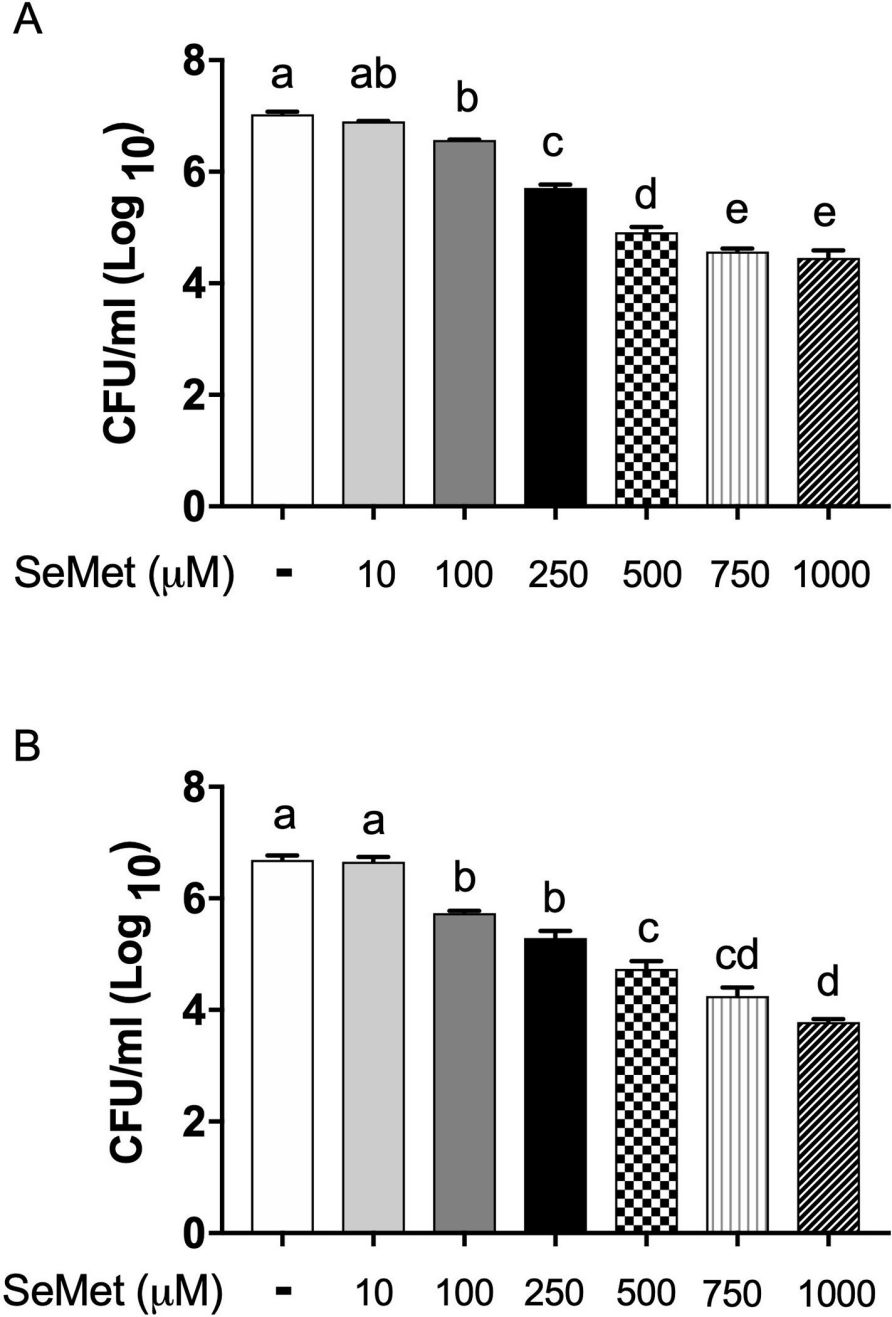
High concentrations of SeMet lead to reduced intracellular and extracellular bacterial burden in infected macrophages. RAW 264.7 cells were infected with *B*. *thailandensis* and treated with varying concentrations of SeMet for 18 hours. (A) Remaining intracellular bacterial burden in macrophages following the 18 hour treatment, quantified through plating serial dilutions of macrophage lysates. Significant differences were assessed by one-way ANOVA, a > b > c > d > e (*P* < 0.05). Results are representative of data from at least three independent experiments with treatment groups run in triplicate wells. (B) Extracellular bacterial burden following the 18 hour treatment, enumerated by plating serial dilutions of collected supernatant. Significant differences were assessed by one-way ANOVA, a > b > c > d (*P* < 0.05). All data are represented as the mean ± standard error of the mean.

We first tested the ability of varied concentrations of SeMet to affect intracellular bacterial burden in *B*. *thailandensis*-infected macrophages by the end of the 18 hour treatment (Fig 1A). SeMet treatments produced a significant dose response with fewer intracellular bacteria at higher concentrations, starting at 100 μM SeMet and plateauing around 750–1000 μM (Fig 1A). The highest SeMet concentration (1000 μM) resulted in over a 2.5 log_10_ unit reduction in intracellular bacterial burden compared to untreated cells.

We had previously seen evidence of a dynamic interplay between the extracellular and intracellular spaces of our macrophage infection model [24], whereby extracellular bacteria could gain entrance to the inside compartments of macrophages, and intracellular bacteria, upon rupture or lysis of the macrophage, could exit back into the extracellular space. Such movement of bacteria between intracellular and extracellular spaces would be predicted based on the known pathogenesis of *Burkholderia* [4, 8]. Although we began the 18 hour treatment with a primarily intracellular infection, we wanted to evaluate the effect of SeMet on the extracellular bacterial burden as well, due to this potential movement of *B*. *thailandensis* between the intracellular and extracellular niches. In evaluating the remaining bacterial burden of the extracellular niche, we found a titratable effect of SeMet such that increasing concentrations of SeMet, above 100 μM, led to further reduction of the extracellular bacterial burden compared to untreated cells (Fig 1B). With 1000 μM SeMet treatment the extracellular bacterial burden, similar to the intracellular bacterial burden, was reduced over 2.5 log_10_ units compared to the untreated control.

### SeMet significantly inhibits bacterial growth and induces filamentation

**Fig 2:**
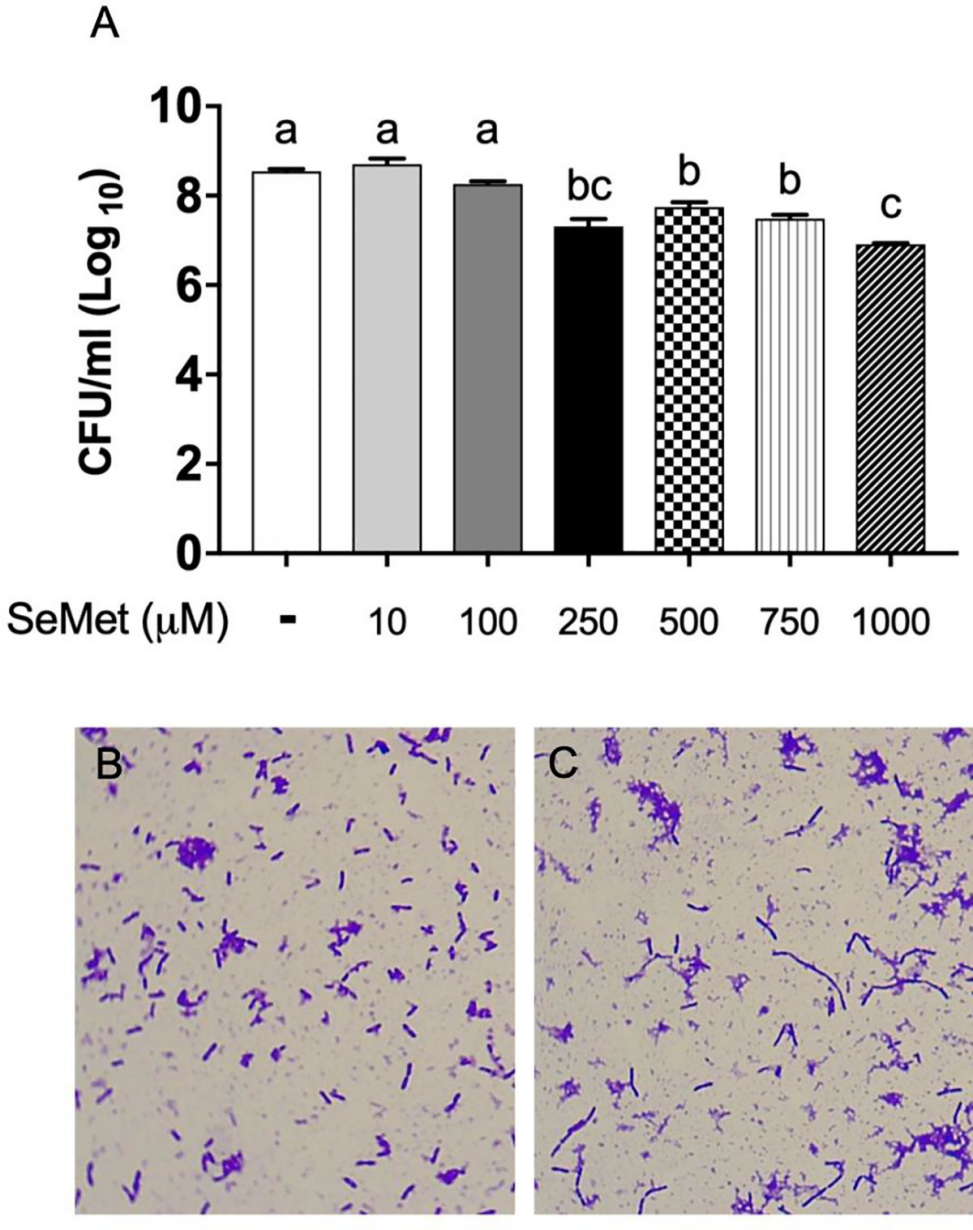
SeMet inhibits *B*. *thailandensis* growth and induces filamentation. *B*. *thailandensis* was grown from a starting concentration of 2 x 10^6^ CFU/ml with varying concentrations of SeMet for 18 hours in the absence of RAW 264.7 macrophages. (A) Remaining viable bacteria quantified by plating serial dilutions. Data are represented as the mean ± standard error of the mean. Significant differences were assessed by one-way ANOVA, a > b > c (*P* < 0.05). (B and C) Samples of bacteria from the untreated control (B) and 1000 μM SeMet treatment (C) were prepared on slides, stained with crystal violet, and imaged at 1000x magnification. All results are representative of data from at least three independent experiments with treatment groups run in triplicate wells. At least one slide was created, stained, and viewed from each treatment group for each of three independent experiments.

After seeing reductions in both intracellular and extracellular bacterial burden in our macrophage infection model, we determined the effect of SeMet alone on the bacteria. We found that increasing SeMet concentrations resulted in increased inhibition of *B*. *thailandensis* growth over the same 18 hour treatment (Fig 2A). With 1000 μM SeMet treatment there was a 1.5 log_10_ unit difference in bacterial density compared to the untreated control. Imaging the bacteria at the end of the 18 hour treatment revealed a filamentous morphology to some of the *B*. *thailandensis* organisms that had been treated with 1000 μM SeMet (Fig 2B and 2C). In exploring this morphological change further, we determined that some *B*. *thailandensis* began to form noticeable filaments at around 250 μM SeMet and that the higher concentrations yielded longer filaments (S1 Fig). Even at 1000 μM SeMet, however, there were still many single, non-filamentous bacteria.

### The concentration of SeMet impacts its ability to enhance the individual effects of IFN-γ and ceftazidime on bacterial burden

**Fig 3:**
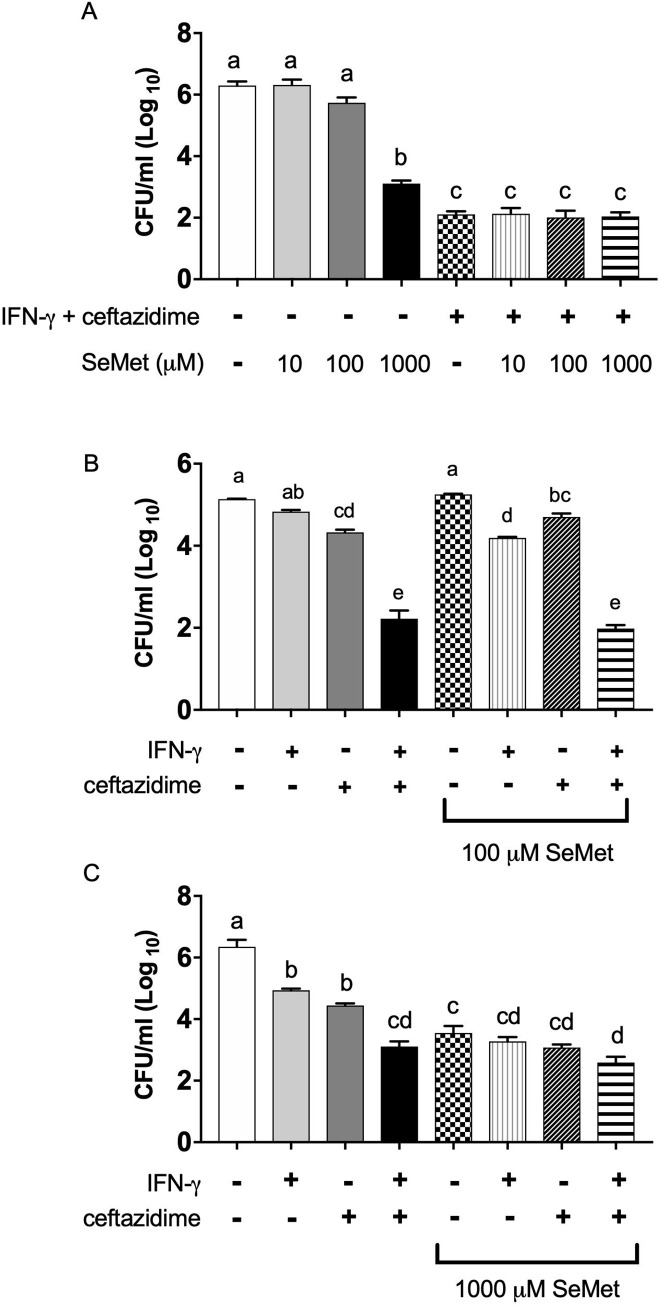
The effects of SeMet on the individual treatments of IFN-γ and ceftazidime during macrophage infection. RAW 264.7 cells were infected with *B*. *thailandensis* and treated with varying concentrations of SeMet and/or the combination of ceftazidime (10 μg/ml) and IFN-γ (10 ng/ml) for 18 hours. Remaining intracellular bacterial burden was then quantified through plating serial dilutions of macrophage lysates. Data are represented as the mean ± standard error of the mean. (A) Intracellular bacterial burden of infected macrophages after treatment with ten-fold dilutions of SeMet alone with the combination of IFN-γ and ceftazidime for 18 hours. Significant differences were assessed by one-way ANOVA, a > b > c (*P* < 0.05). (B) Intracellular bacterial burden of infected macrophages after treatment with 100 μM SeMet alone or in combination with the individual treatments of IFN-γ and ceftazidime. Significant differences were assessed by one-way ANOVA, a > b > c > d > e (*P* < 0.05). (C) Intracellular bacterial burden of infected macrophages after treatment with 1000 μM SeMet alone or in combination with the individual treatments of IFN-γ and ceftazidime. Significant differences were assessed by one-way ANOVA, a > b > c > d (*P* < 0.05). All results are representative of data from two (A and B) or three (C) independent experiments with treatment groups run in triplicate wells.

Having established an impact of SeMet on both the intracellular and extracellular spaces of the infection model, and having identified the ability of SeMet to inhibit bacterial growth, we next wanted to compare its effect to the known beneficial treatments of IFN-γ and ceftazidime alone or in combination. We first tested ten-fold dilutions of SeMet on infected macrophages alone or in addition to the combination of IFN-γ and ceftazidime (Fig 3A). As we had seen before, the lower concentrations of SeMet had a negligible effect on the intracellular bacterial burden by the end of the 18 hour treatment and only the 1000 μM SeMet treatment resulted in a large and statistically significant reduction. Surprisingly, the effect of 1000 μM SeMet alone was almost comparable to the reduction achieved by the combination of IFN-γ and ceftazidime. On average, the 1000 μM SeMet resulted in over a 2.5 log_10_ unit reduction in intracellular bacterial burden compared to the untreated control, while the combination of IFN-γ and ceftazidime resulted in over a 3.5 log_10_ unit reduction in intracellular bacteria. The addition of SeMet to the combination of IFN-γ and ceftazidime did not result in further reduction of the intracellular bacterial burden for any of the SeMet concentrations tested.

Next, we separated IFN-γ from ceftazidime and determined the effect of SeMet when combined with each treatment individually. Separately, both IFN-γ and ceftazidime led to significant reductions of intracellular bacterial burden in *B*. *thailandensis* infected macrophages (Fig 3B), as seen previously [24]. At 100 μM, SeMet treatment resulted in no reduction of intracellular bacterial burden by itself, and significantly added to the effect of IFN-γ but not ceftazidime. The addition of 1000 μM SeMet further reduced the bacterial burden of IFN-γ- as well as ceftazidime-treated cells alone by an additional 1.5 log_10_ units, although the resulting bacterial burden was indistinguishable from the effect of SeMet alone (Fig 3C).

### High concentrations of SeMet lead to protected membrane integrity of macrophages during infection

**Fig 4:**
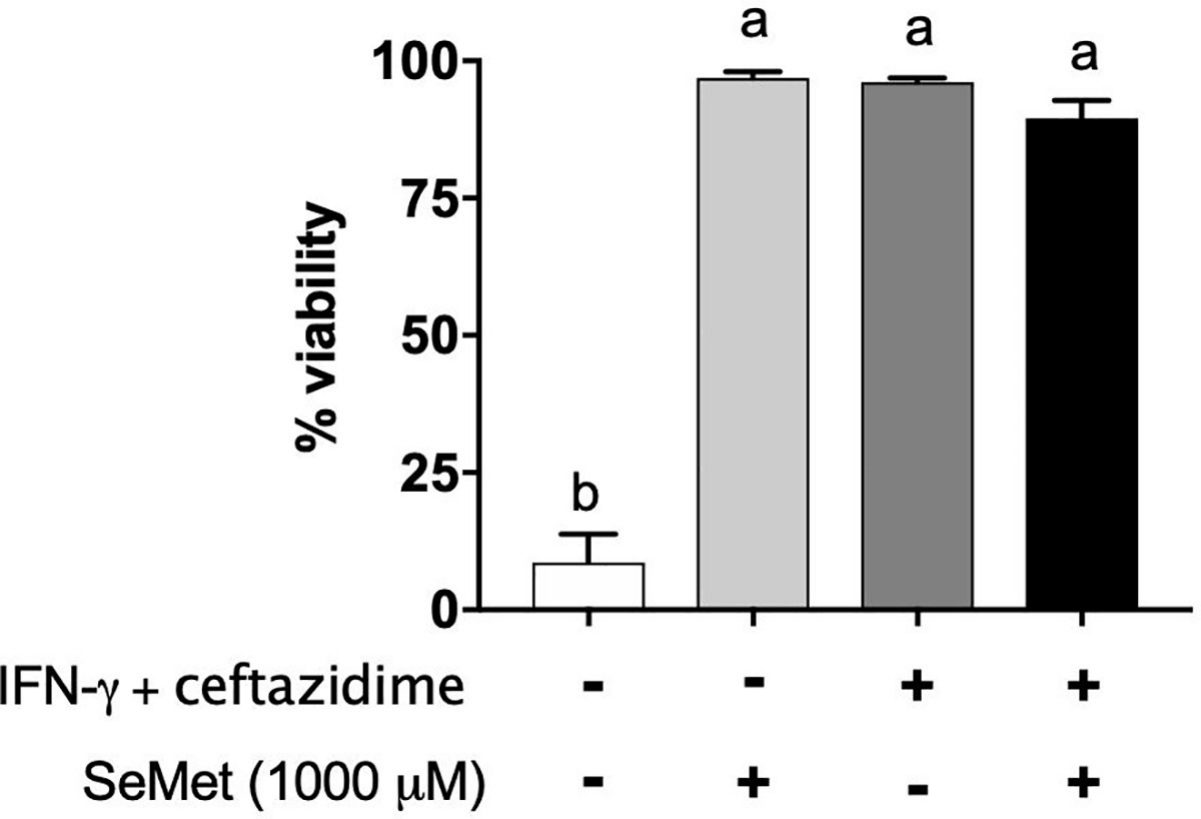
SeMet helps preserve membrane integrity of infected macrophages. RAW 264.7 cells were infected with *B*. *thailandensis* and treated with SeMet (1000 μM) and/or the combination of ceftazidime (10 μg/ml) and IFN-γ (10 ng/ml) for 18 hours. Survival was assessed by assessing membrane integrity through trypan blue exclusion at 18 hours post-treatment. Data are represented as the mean ± standard error of the mean. Significant differences were assessed by one-way ANOVA, a > b (*P* < 0.05). Results are representative of data from at least two independent experiments with treatment groups run in triplicate wells, and five random fields of view imaged and counted per well.

Viability of *B*. *thailandensis* infected macrophages was assessed by determining membrane integrity by trypan blue exclusion at 18 hours post-treatment with 1000 μM SeMet. Infected macrophages that were left untreated showed significant damage, with fewer than 10% surviving by the end of 18 hours (Fig 4). On the other hand, the presence of SeMet during macrophage infection led to 95% macrophage survival within the same time frame, similar to the IFN-γ and ceftazidime combination treatment group. The combination of all three treatments did not result in higher survival rates.

## Discussion

We had previously shown that treatment of *B*. *thailandensis* infected macrophages with ceftazidime and IFN-γ resulted in a synergistically reduced intracellular bacterial burden which was 3 to 4 log_10_ units lower than untreated controls at the end of an 18 hour treatment. The effect was partly attributed to increased intracellular ROS, due to IFN-γ stimulation, as the antioxidants GSH and NAC reversed the IFN-γ effect on bacterial burden [24]. Further evidence of a role for ROS in the effect of IFN-γ and ceftazidime was that the pro-oxidant drug L-buthionine sulfoximine increased levels of intracellular ROS by itself, and when combined with ceftazidime resulted in a synergistic reduction of the intracellular bacterial burden [24].

After observing the reversal of the IFN-γ and ceftazidime effect with two cellular antioxidants, GSH and NAC, we asked whether other antioxidants would also reverse the effectiveness of the IFN-γ treatment and further corroborate the influence of ROS in its bactericidal activity. We therefore investigated the role of SeMet in our macrophage infection model. To our surprise, treatment of infected macrophages with high concentrations of SeMet alone led to large reductions of intracellular bacteria by the end of the 18 hour treatment period. In fact, the resulting bacterial burden from 1000 μM SeMet alone was nearly comparable to the combinatorial effect of IFN-γ and ceftazidime (Fig 3C). High concentrations of SeMet also resulted in significant reductions to the extracellular bacterial burden, though this result was less surprising after learning of SeMet’s impact on the intracellular space. Assuming that the bacteria could move between the compartments during the infection as we had previously hypothesized [24], we could have predicted that a reduction in intracellular bacteria would eventually affect the extracellular compartment too, and vice versa. It is likely that these large reductions in bacterial burden also contributed to the preservation of membrane integrity of SeMet treated macrophages compared to the untreated controls.

Evidence from previous studies suggested that the increased ROS, generated internally due to IFN-γ stimulation of macrophages, was at least partly responsible for the bactericidal effect of the IFN-γ and ceftazidime combination treatment on infected macrophages [24]. If SeMet was acting as an antioxidant, either directly by scavenging radicals, or indirectly through increased production of glutathione peroxidases, we would have expected some abolishment of the IFN-γ effect leading to an increased bacterial burden with the combination of SeMet and IFN-γ, compared to IFN-γ alone. That is what we had seen previously with the antioxidants NAC and GSH [24]. Instead we observed the opposite effect—low concentrations of SeMet significantly *enhanced* the IFN-γ effect (Fig 3B) and even high concentrations failed to reverse the IFN-γ effect, suggesting that the mechanism of action of SeMet may not be directly related to scavenging the IFN-γ induced ROS. SeMet could, however, be acting directly as an antioxidant in a different compartment from the IFN-γ induced ROS, though it would beg the question why GSH and NAC, which are also antioxidants and precursors to glutathione peroxidases, have different effects than SeMet? Alternatively, SeMet could also be acting in numerous non-antioxidant roles through the incorporation of selenium into various metabolic pathways or through other mechanisms explored below.

Although surprising that SeMet did not directly counteract the IFN-γ effect, a more surprising observation was the magnitude of the effect of SeMet, by itself, to reduce intracellular bacterial burden (over a 2.5 log_10_ unit reduction) compared to the untreated control, surpassing the effect of the antibiotic by itself and reducing the bacterial burden to a level comparable to the combination of the immune stimulant and antibiotic treatment (Fig 3C). Although more research is needed to understand the full mechanism by which SeMet exerts such a large effect during the macrophage infection, we did show a role for SeMet in inhibiting the growth of *B*. *thailandensis*. By inhibiting bacterial growth, SeMet would be expected to reduce overall extracellular bacterial numbers as well as prevent increases in intracellular numbers, which is precisely what we observed.

We are not the first to show that selenium can lead to a bacteriostatic inhibition of growth. Several studies have also shown inhibition of bacterial growth with selenium or selenium-containing nanoparticles [34] at concentrations both above [35] and below [36] the concentrations used here. Aribi et al. showed that selenium supplementation of media led to inhibited growth of *Staphylococcus aureus*, but this effect was seen with 20 ng/ml (~0.1 μM) of sodium selenite, about a 10,000x lower concentration than tested here [36]. They also observed varied effects at both higher and lower concentrations whereas we observed a consistent dose-dependent inhibitory effect within a wide range of concentrations (250–1000 μM). In another study, selenium deficient mice had an increased susceptibility to infection, and exhibited a greater bacterial burden compared to control mice [37], suggesting the overall importance of selenium status in helping to fight bacterial disease.

Although SeMet inhibition of bacterial growth helps to explain the reduced bacterial burden in SeMet treated macrophages, it does not seem sufficient to explain the entire effect, since the effect of SeMet on infected macrophages was twice the magnitude of the effect of SeMet on bacteria alone (almost a 3 log_10_ unit reduction of intracellular bacteria vs. a 1.5 log_10_ unit inhibition of bacterial growth) within the same time frame. Recognizing the movement of bacteria between the extracellular and intracellular environments, we cannot completely rule out the possibility that a 1.5 log_10_ unit inhibition of bacterial growth in the extracellular environment could, throughout the 18 hour treatment, result in a much larger reduction in intracellular bacterial burden. However, another consideration is that 100 μM SeMet, while not significantly inhibiting bacterial growth itself (Fig 2) added to and significantly enhanced the IFN-γ effect on reduced intracellular bacterial burden (Fig 3B), suggesting that SeMet may have an additional effect besides simply inhibiting growth. For example, SeMet may stimulate increased phagocytosis of bacteria and clearance of the internalized bacteria from the macrophages. Others have shown that low concentrations of selenium (~0.1 μM and 0.1 ppm) can increase phagocytosis [36, 38], though it remains to be seen if higher concentrations may also have this effect.

Another explanation of the SeMet effect may involve the filamentous morphology of *B*. *thailandensis* that was observed in some bacteria after exposure to 250+ μM SeMet (Fig 2C and S1 Fig). Filamentation results when bacteria continue to grow without septation into separate organisms, and can be induced by many chemical and physical stressors such as antimicrobials or starvation [39–42]. It is a well-documented phenomenon for *Burkholderia* and several other Gram-negative pathogens upon exposure to beta-lactam antibiotics [43, 44], such as ceftazidime. A study conducted with the closely related *B*. *pseudomallei* showed that filamentous bacteria, formed through exposure to antibiotics, had decreased motility and a decreased ability to lyse host monocytic cells [39]. Altered virulence of the SeMet-induced filamentous *B*. *thailandensis* could explain the lack of damage to macrophages in SeMet treatment groups compared to untreated controls seen in Fig 4, though further studies are required to determine if the SeMet induced filaments mimic antibiotic-induced filaments in terms of altered virulence. On the other hand, filamentation may have resulted in a slight underestimation of the true bacterial burden in our studies, since a single filament is likely to give rise to just one CFU on agar [45], but would otherwise have the potential to divide into its numerous bacterial components after the removal of the stress in vitro [39]. However, we do not yet know if SeMet induced filaments behave in the same ways as antibiotic-induced filaments in their reversion to single bacteria. In one study, bacterial filaments that had stalled cell division for a lengthy time could never again initiate division, showing that filament reversion to single bacteria is not always possible [46]. Regardless, since filamentous bacteria only made up a fraction of the bacteria exposed to SeMet and observed on the stained slides in Fig 2 and S1 Fig, we predict the filamentation effect to be minor.

In regards to the preserved membrane integrity of SeMet treated macrophages, the decreased bacterial burden within those cells likely contributed to the health of their membranes, though it was surprising that SeMet treated cells had a significantly greater intracellular bacterial load compared to the IFN-γ and ceftazidime treated cells, and yet had equal viability to the IFN-γ and ceftazidime treated group. This suggests that another effect of SeMet may also be at play, perhaps related to levels of inflammation induced in the infected cells. Related to this, another interesting finding was that high concentrations of SeMet, well beyond the concentrations that will normally cause toxicity to healthy macrophages, were actually protective towards the membrane integrity of the macrophages during the *B*. *thailandensis* infection. Selenium is an essential trace element but is required in very low concentrations (less than 1 μM in human plasma) [31] and excess selenium intake can lead to toxicity [28, 31]. A previous study found that sodium selenite was toxic to healthy RAW 264.7 macrophages, the same cell line used in the current study, at a concentration of just 128 μM [47]. In that study, fewer than 10% of macrophages remained viable after a 12 hour incubation as measured by MTT assay [47]. In contrast, 97% of our *infected* RAW 264.7 macrophages remained viable, with membranes intact, after 18 hour treatment with a concentration almost 10x as high, measured by trypan blue exclusion. That the same nutrient could be toxic or protective depending on infection state of the host cells seems paradoxical, but this too is supported by other studies. Human supplementation with selenium at doses well in excess of the tolerable upper intake level (400 μg/day) [48] were shown to decrease mortality rates in people suffering from sepsis [49], which in agreement with the results seen in the present study might suggest an increased need for and tolerance of selenium during times of bacterial infection. Selenium has also been shown to have anti-inflammatory properties and prevent immunopathology due to inflammatory signaling cascades [28, 47, 50–53], which might help explain the lack of damage and preserved viability of infected macrophages treated with SeMet. Lastly, since trypan blue exclusion is unable to distinguish between healthy cells and apoptotic cells, we cannot discount the possibility that the SeMet treated macrophages were dying by apoptosis, though if they were, it would likely be preferable to the pro-inflammatory mechanisms of cell death associated with *Burkholderia* [54–56]. We intend to look further into different types of cell death in future work.

In conclusion, we have shown that treating *B*. *thailandensis* infected macrophages with high concentrations of SeMet led to better overall macrophage health including reductions in both intracellular and extracellular bacterial burden as well as the preservation of membrane integrity. The decreased bacterial burden due to SeMet treatment may be partially explained by its inhibition of rapid growth of *B*. *thailandensis*, though other explanations such as an increase in phagocytosis or altered virulence of filamentous forms have not yet been ruled out. The observation that high concentrations of SeMet preserved the membrane integrity of infected macrophages could be due to the decreased intracellular bacterial burden within those cells, but could also be due to other factors, explored above, such as altered bacterial virulence or a decrease in inflammatory cytokines and signaling during infection. Regardless of the mechanism, we provide another piece of evidence to suggest that high dose selenium treatments may be beneficial during infected states. If this were a more broadly seen effect with selenium compounds, it would change the way initial toxicity studies are analyzed, and how final concentrations are chosen to move forward in experimental models. Further research is needed to understand if SeMet would be protective towards macrophages during infection by other bacterial pathogens, and also whether high doses of other selenium compounds, or other antioxidants entirely, may have a similar protective effect under the same infection conditions. The ongoing work in our lab aims to better understand the roles of antioxidants, especially selenium compounds, in bacterial infection models.

## Supporting information

S1 File Raw data for Figs 1–4 and S1 Fig. Individual values that make up each mean.
(PDF)

S1 Fig High concentrations of SeMet induce filamentous morphology in *B. thailandensis*. (A-G) *B*. *thailandensis* was grown from a starting concentration of 2 x 10^6^ CFU/ml with varying concentrations of SeMet for 18 hours. Remaining viable bacteria were then quantified through plating of serial dilutions, and 10 μl samples of bacteria from SeMet treated wells were prepared on slides, heat-fixed, stained with crystal violet, and imaged at 1000x magnification. Images are representative of at least two independent experiments and from a total of 4 wells for each concentration of SeMet. At least one slide was created, stained, and viewed from each treatment well.
(JPG)
