# Supplementary material for: Dietary antioxidant seleno-L-methionine protects macrophages infected with Burkholderia thailandensis
Source: PLoS One. 2020 Sep 3;15(9):e0238174. doi: 10.1371/journal.pone.0238174 (PMC7470333; doi:10.1371/journal.pone.0238174)
Supplement: S1 File — Individual values that make up each mean. (PDF) [file pone.0238174.s001.pdf]

S1 File. Raw data for figures 1-4. Individual values that make up each mean.

Figure 1A Data: CFU/ml (log10 scale)

Untreated	10 µM	100 µM	250 µM	500 µM	750 µM SeMet	1000 µM SeMet
7.1173	6.9191	6.5798	5.5911	4.9294	4.6128	4.7160
7.0294	6.8976	6.5682	5.7853	4.7404	4.6335	4.3617
6.9345	6.8865	6.5682	5.7482	5.0719	4.4624	4.3010

Figure 1B Data: CFU/ml (log10 scale)

Untreated control	10 µM SeMet	100 µM SeMet	250 µM SeMet	500 µM SeMet	750 µM SeMet	1000 µM SeMet
6.5563	6.5798	5.6532	5.4771	4.4624	3.9542	3.7404
6.8129	6.5563	5.7634	5.3424	4.8195	4.4771	3.7324
6.7076	6.8325	5.7853	5.0414	4.9243	4.3222	3.8865

Figure 2 Data: CFU/ml (log10 scale)

Untreated control	10 µM SeMet	100 µM SeMet	250 µM SeMet	500 µM SeMet	750 µM SeMet	1000 µM SeMet
8.4914	8.4624	8.3222	7.6335	7.5441	7.6335	6.9345
8.4914	8.8325	8.3222	7.0792	7.9191	7.3424	6.8692
8.6532	8.8261	8.1461	7.2304	7.7634	7.4771	6.9345

Figure 3A Data: CFU/ml (log_{10} scale)

Untreated control	10 µM SeMet	100 µM SeMet	1000 µM SeMet	IFN-gamma + ceftazidime	10 µM SeMet + IFN-gamma + ceftazidime	100 µM SeMet + IFN-gamma + ceftazidime	1000 µM SeMet + IFN-gamma + ceftazidime
6.0414	6.5682	5.8921	3.3010	2.3010	2.1761	1.6021	1.7782
6.5051	6.3979	5.3802	2.9542	1.9542	2.4314	2.3222	2.1761
6.3424	5.9912	5.9294	3.0792	2.0792	1.7782	2.1139	2.1761

Figure 3B: CFU/ml (log10 scale)

Untreated control	IFN-gamma	ceftazidime	IFN-gamma + ceftazidime	100 µM SeMet Control	100 µM SeMet + IFN- gamma	100 µM SeMet + ceftazidime	100 µM SeMet + ceftazidime + IFN-gamma
5.2304	4.6990	4.5911	2.3424	5.5315	4.3802	4.6990	1.6990
5.0792	4.8633	4.6532	2.4472	5.3617	4.0414	4.7160	1.6990
5.4150	4.8451	4.6232	2.3222	5.4624	4.1461	4.6435	2.1139

Figure 3C Data: CFU/ml (log10 scale)

Untreated control	IFN-gamma	ceftazidime	IFN-gamma + ceftazidime	1000 µM SeMet Control	1000 µM SeMet + IFN-gamma	1000 µM SeMet + ceftazidime	1000 µM SeMet + ceftazidime + IFN-gamma
6.2041	4.9191	4.5563	3.2553	3.8751	3.3802	2.8808	2.5051
6.0414	4.8513	4.3222	2.7709	3.6628	3.0000	3.1139	2.9494
6.7993	5.0414	4.4472	3.3010	3.1139	3.4472	3.2304	2.3222

Figure 4 Data: % viability

Control	1000 µM SeMet	IFN-gamma + ceftazidime	1000 µM SeMet + IFN-gamma + ceftazidime
0	96.9	98.1	85.8
0	99	94.9	67.8
0	81.4	92.2	97.4
16.7	96.8	96.2	83.9
0	96.1	97.4	92
21.1	100	93.7	99.2
0	93.1	99.2	90.3
0	98.1	97.1	55.3
0	100	96	99.3
0	99	97.6	96.5
0	97.6	99.1	95.2
0	97.6	95.7	95.2
0	100	98.6	95.2
16.7	96.9	97.3	93
75	100	88.8	97.6
