# Supplementary figures and images for: Dietary antioxidant seleno-L-methionine protects macrophages infected with Burkholderia thailandensis
Source: PLoS One. 2020 Sep 3;15(9):e0238174. doi: 10.1371/journal.pone.0238174 (PMC7470333; doi:10.1371/journal.pone.0238174)

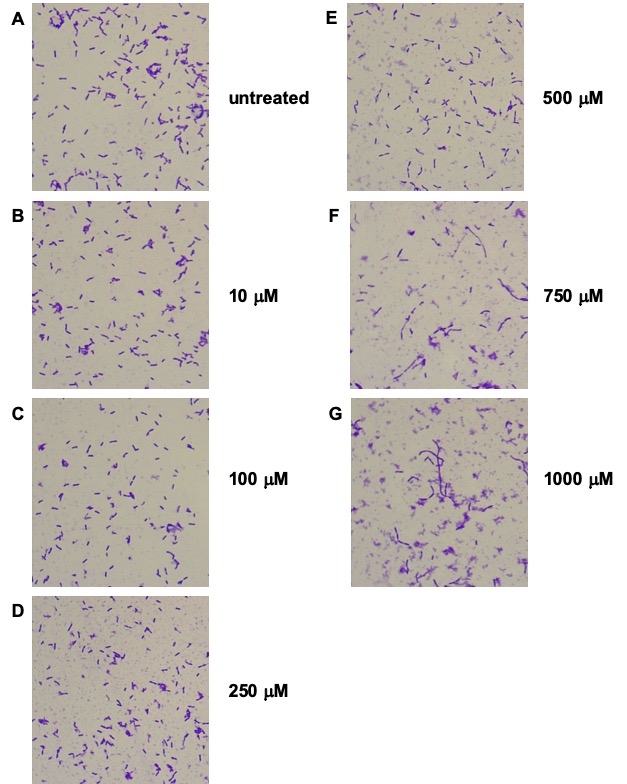

Supplement: S1 Fig — (A-G) B. thailandensis was grown from a starting concentration of 2 x 10^6^ CFU/ml with varying concentrations of SeMet for 18 hours. Remaining viable bacteria were then quantified through plating of serial dilutions, and 10 μl samples of bacteria from SeMet treated wells were prepared on slides, heat-fixed, stained with crystal violet, and imaged at 1000x magnification. Images are representative of at least two independent experiments and from a total of 4 wells for each concentration of SeMet. At least one slide was created, stained, and viewed from each treatment well. (JPG) [file pone.0238174.s002.jpg]
